# Transcriptional Analysis of Cell Division-Related Genes in *Weizmannia coagulans* BC99 Under Low pH Conditions

**DOI:** 10.3390/microorganisms14040839

**Published:** 2026-04-08

**Authors:** Yanqi Zhang, Pengyan Li, Lijuan Wang, Jianrui Sun, Shanshan Tie, Ying Wu, Dahong Wang, Jie Zhang, Shaobin Gu

**Affiliations:** 1 College of Food and Bioengineering, Henan University of Science and Technology, Luoyang 471023, China; 2 College of Basic Medical Science, Ningxia Medical University, Yinchuan 750004, China; 3 Henan Engineering Research Center of Food Microbiology, Luoyang 471023, China; 4 National Demonstration Center for Experimental Food Processing and Safety Education, Luoyang 471023, China; 5 Henan University-Enterprise R&D Center for Scientific Evidence-Based and Industrialization Application of Probiotics, Luoyang 471023, China

**Keywords:** cell division, *Weizmannia coagulans* BC99, probiotics, acid stress, proton motive force, transcriptome

## Abstract

Environmental pH plays a critical role in microbial fermentation processes. *Weizmannia coagulans* attracts particular attention for exceptional acid tolerance and lactic acid productivity. Yet acidic stress impacts on its cell division regulation remain unclear. Here, a critical pH value (pH 4.20) for growth inhibition of the Gram-positive bacterium *Weizmannia coagulans* strain BC99 was first established. Transcriptomic analysis of metabolic pathways was then performed. The multi-layered regulatory network underlying acid stress-induced cell division was elucidated. Integrated transcriptomic and physiological analyses reveal that acid stress triggers multigene expression reprogramming. This drives core metabolic network reorganization, coordinately regulating division processes. RNA-seq analysis demonstrated acid stress triggered differential expression of division genes (FtsZ/Q downregulation), ATP synthase suppression, and peptidoglycan transport reduction, while enhancing membrane rigidification (*Cfa*) and magnesium homeostasis (*CorA*). The PhoPR dual-component system emerged as a central regulator, inhibiting septal assembly via RipA hydrolase and RpsU ribosomal suppression while rerouting carbon flux to glycolysis, elucidating bacterial acid adaptation mechanisms. Collectively, these adaptive changes prioritize cell survival over active proliferation under acidic conditions. This study provides molecular insights into how *W. coagulans* preserves viability under acid stress, offering a theoretical basis for optimizing its performance in probiotic applications.

## 1. Introduction

*Weizmannia coagulans*, a Gram-positive, non-pathogenic bacterium, exhibits significant probiotic properties and substantial industrial application potential [1]. This bacterium forms spores ensuring survival in harsh gastrointestinal tract conditions. Upon intestinal lumen entry, spores germinate and proliferate. This will help modulate microbiota, enhance barrier integrity, and exert immunomodulatory effects [2,3]. *W. coagulans* is widely recognized as the “King of Probiotics” owing to its exceptional thermal tolerance, with an optimal growth temperature range of 40–45 °C, and its unique capacity to produce L-lactic acid exclusively via the homofermentative pathway [4,5]. Current research has elucidated the regulatory pathways associated with spore formation and germination; however, understanding of the underlying cell division mechanisms remains limited.

pH is a critical environmental parameter in microbial fermentation processes, exerting profound influences on cellular membrane structure and permeability, the activity of key enzymes, and the allocation of metabolic fluxes, while also directly modulating bacterial proliferation and cell division [6]. Bacteria exposed to external acid stress adjust saturated versus unsaturated fatty acid ratios in membrane phospholipids. This modification enhances membrane fluidity. It preserves nutrient absorption and energy-metabolism efficiency [7]. Microorganisms require intracellular pH homeostasis for cell division. They employ Na^+^/H^+^ and K^+^/H^+^ antiporters plus F_1_F_0_-ATPase proton pumps. These systems jointly mediate excess proton efflux, stabilizing the intracellular microenvironment [8]. Low external pH indirectly supports cell division by remodeling energy metabolism. Acidic conditions enhance cytochrome bd oxidase activity. This elevates respiratory chain function, improving ATP synthesis efficiency and supplying adequate energy for cell division [9].

At the molecular level, low pH exerts a direct regulatory influence on the assembly of the FtsZ-dependent Z-ring, the central macromolecular complex governing bacterial cell division [10]. Studies have demonstrated that the inhibitory effect of MinC on FtsZ polymerization is highly dependent on pH, and this inhibition can be effectively counteracted by the ZapA protein [11]. Under acidic conditions, MinC activity may be attenuated, resulting in aberrant localization or reduced stability of the Z-ring and thereby leading to misplaced septation sites and diminished division efficiency. FtsZ constitutes a core prokaryotic cytoskeletal element. Its GTPase activity, polymerization dynamics, and interactions with divisome proteins (FtsA, SepF, EzrA) are strongly influenced by intracellular pH [12]. This governs protein conformation and surface charge distribution. Consequently, environmental acidification can perturb this finely tuned regulatory network, prompting adaptive cellular responses such as the upregulation of genes including *zapA*, *ftsA*, *sepF*, and *ezrA* to compensate for impaired Z-ring function and preserve division fidelity [13,14]. Transcriptional responses undergo coordination and amplification through two-component systems (TCS) or sigma factor-mediated signaling pathways. This facilitates balanced allocation of cellular resources among cell division, stress adaptation, and secondary metabolic processes.

Although these mechanisms have been well characterized in model Gram-positive bacteria, the key regulatory mechanisms underlying the entry of *W. coagulans* cells into growth arrest under acidic stress remain poorly understood. This knowledge deficit hinders the rational engineering of *W. coagulans* for synthetic biology applications and limits the development of precision probiotic strategies. Therefore, this study aims to elucidate the regulatory mechanism of cell division in *W. coagulans* under low-pH conditions and delineate its unique division control network, laying a solid theoretical foundation for improving the industrial fermentation performance and probiotic efficacy of *W. coagulans*.

## 2. Materials and Methods

### 2.1. Reagents and Materials

Glucose (purity ≥ 98%) was purchased from Tianjin Kemiou Chemical Reagent Co., Ltd. (Tianjin, China). Yeast extract powder, tryptone were purchased from Beijing Aobosun Biotechnology Co., Ltd. (Beijing, China). All other reagents were purchased from Tianjin Deen Co., Ltd. (Tianjin, China).

### 2.2. Microorganism and Culture Condition

*Weizmannia coagulans* BC99 (*Heyndrickxia coagulans* strain: BC99) was provided by Wecare Probiotics Co., Ltd. (Suzhou, China). The accession number in NCBI is SAMN26203093. The medium used in this study consists of 15.0 g/L glucose, 15 g/L tryptone, 10 g/L yeast extract powder, and 5 g/L MgSO_4_, with a pH of 7.00; *W. coagulans* BC99 was inoculated in 25 mL of liquid medium with 2% inoculation amount at 37 °C and 180 rpm for 24 h, and all the inoculated strain were those that had been activated twice consecutively and OD_600_ > 0.8.

### 2.3. Determination of the Critical pH

Lactic acid (2 M) was used to adjust the pH of the medium to 4.10, 4.20, 4.30, 4.40, and 4.50, respectively. Cultures were incubated as described in Section 2.2. Samples were collected at 2 h intervals from 0 to 24 h, and the OD_600_ was measured using a microplate reader. The specific growth rate was calculated from Equation (1):

**µ**N = dN/dt (1)

In the formula: μ—specific growth rate of cells (/h);

N—cell concentration (CFU/mL);

t—cell culture time (h).

Data from three biological replicates were used for analysis. According to the change in culture time, growth curves and the curve of the number of culturable bacteria at different initial pH values on the culturability and proliferation of the bacteria was analyzed using the plate counting method.

### 2.4. Determination of Carbon and Nitrogen Source Consumption

The consumption of carbon sources is indicated by the change in glucose content. The determination method is based on the approach described by Yue et al. [15]. *W. coagulans* BC99 cells were cultured as described in Section 2.2. A total of 10 mL were cultured to 0. The culture medium at 2 h, 4 h, 6 h, and 8 h was centrifuged to remove the supernatant, and then filtered to remove bacteria to obtain the cell-free supernatant. The glucose concentration in the supernatant was determined. Data from three biological replicates were used for analysis.

The variation in nitrogen source content is indicated by the changes in total amino acids. Sampling times and processing methods were determined as shown above. The amino acid content in the culture medium was quantitatively determined using the amino acid detection kit provided by Beijing Solbo Biotechnology Co., Ltd. (Beijing, China).

### 2.5. Transcriptomic Analysis of Strain BC99 Under Low-pH Treatment

#### 2.5.1. RNA Isolation, Library Construction, and Sequencing

RNA was extracted from bacteria in the logarithmic growth phase. TRIzol^®^ Reagent (Invitrogen, Carlsbad, CA, USA) was used to extract total RNA from the bacterial samples, and its concentration, purity and integrity (RIN ≥ 8) were strictly assessed by Nanodrop, agarose gel electrophoresis and Agilent 2100 bioanalyzer to ensure that the quality of the starting material for library was up to standard (total amount ≥ 2 μg, OD_260/280_ between 1.8 and 2.2). Subsequently, given the lack of polyA tails in prokaryotic mRNAs, Illumina TruSeq™ Stranded Total RNA Library Prep Kit (Illumina, San Diego, CA, USA) was used for library construction. rRNA was specifically removed by Ribo-Zero Magnetic kit (EpiCentre, Madison, WI, USA) to enrich mRNA. The enriched mRNA was fragmented into a small fragment of about 200 bp, followed by reverse transcription to synthesize one-strand cDNA with random primers, and dUTP was used instead of dTTP to achieve strand specificity when synthesizing two-strand cDNA. Double-stranded cDNA was end repaired, ‘A’ tail was added, and Y-linker was ligated. The library was amplified and enriched by PCR. Finally, the libraries were bridge-PCR amplified on an Illumina NovaSeq 6000 platform (Illumina, CA, USA) to generate clusters, and high-throughput sequencing was performed using the PE150 strategy to obtain high-quality double-end sequencing data for subsequent bioinformatics analysis.

#### 2.5.2. RNA-Seq Data Analyses

Firstly, the raw sequencing data were strictly quality control and filtered to obtain high-quality clean data. Subsequently, HISAT2 was used to align the valid sequences to the reference genome (GenBank: CP092692.1) for gene mapping and quantification. Following generation of the gene expression matrix, experimental reproducibility and intergroup heterogeneity were assessed through inter-sample correlation analysis and principal component analysis (PCA). On the other hand, systematic functional annotations were conducted for all the genes by using multiple functional databases (e.g., NR, GO, KEGG). The core analysis focused on the screening of differentially expressed genes. DESeq2 was used to identify the significantly changed transcripts among different treatment groups, and GO functional enrichment analysis and KEGG pathway enrichment analysis were used to further elucidate the potential biological functions and regulatory pathways of these differentially expressed genes. By calculating the linear fold change (FC) of the related genes in the experimental group versus the control group, significant changes in genes were identified. In addition, the analysis according to GAO, F. et al. method [16] was extended to the identification of srnas, expression analysis and gene structural features (such as operon and utr), thereby comprehensively reveal the transcriptome dynamics and regulatory network of *W. coagulans* from multiple dimensions under specific conditions.

### 2.6. Reverse Transcription Real-Time Quantitative PCR (RT-qPCR) Validation

To validate the reliability of differentially expressed genes identified through RNA-seq analysis, eight genes closely related to cell division and energy metabolism were selected for validation via RT-qPCR. Reverse transcription was performed using PrimeScript^TM^ RT Master Mix (TaKaRa, RR036A, Paris, France), and the generated cDNA served as the template for qPCR amplification. All qPCR reactions were conducted on a LightCycler^®^ 96 real-time PCR system using FastStart Universal SYBR Green Master (Roche, 04913850001, Basel, Switzerland). The relative expression levels of target genes were determined using the comparative Ct method (2^−ΔΔCt^), with 16S rRNA employed as the internal reference gene for normalization.

### 2.7. Statistical Analysis

To ensure reliable results, all experiments were carried out three times independently. The data are reported as the mean value accompanied by the standard deviation. Statistical analysis of mean differences was conducted using one-way ANOVA in SPSS 25.0 software (USA), with significance set at *p* < 0.05.

## 3. Results

### 3.1. The Influence of Low pH on the Fermentation Performance of W. coagulans

**Figure 1:**
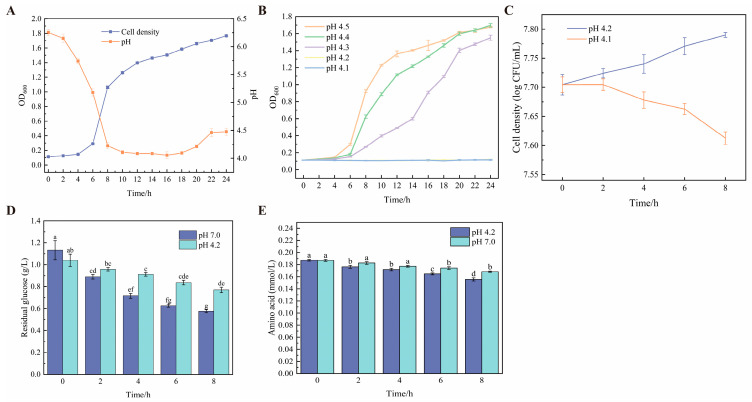
(**A**). The growth curve of *W. coagulans* and the changes in pH during the cultivation process; (**B**). determination of critical pH; (**C**). determinability of cultivation capability; (**D**). the influence of different pH values on glucose consumption; (**E**). the influence of different pH values on nitrogen source consumption. Different letters in (**D**,**E**) indicate significant differences (*p* < 0.05).

According to the growth curve of *W. coagulans* BC99 (Figure 1A), the specific growth rate of the cells markedly declined starting at the late logarithmic phase and approached 0 h^−1^ by 12 h, coinciding with a decrease in medium pH to 4.10–4.50. To evaluate the impact of this pH range on cell viability, the initial pH of the culture medium was adjusted to 4.10–4.50 using lactic acid, and the results are presented in Figure 1B. At initial pH values of 4.30, 4.40, and 4.50, the maximum specific growth rates reached 0.27 ± 0.02 h^−1^, 0.54 ± 0.03 h^−1^, and 0.57 ± 0.01 h^−1^, respectively, which were significantly higher than those observed under other pH conditions (*p* < 0.05). Although these values remained below the maximum specific growth rate recorded during the logarithmic growth phase (0.64 ± 0.02 h^−1^), cell viability remained largely preserved (Figure 1B). The observed decrease in pH coinciding with growth arrest during standard fermentation (Figure 1A) prompted systematic determination of the direct physiological impact exerted by this pH range on bacterial proliferation. In media with initial pH values of 4.20 and 4.10, distinct trends in cell concentration were observed: at pH 4.20, culturable cell counts remained stable, indicating minimal impact on viability; in contrast, at pH 4.10, the culturable cell count steadily declined, suggesting that this condition impairs the maintenance of cell viability (Figure 1C). Therefore, pH 4.20 is considered the critical threshold for sustaining cell viability.

As shown in Figure 1D, in critical pH medium (initial pH 4.20), residual glucose concentration exhibited gradual decline, resulting in final consumption of 10.3 ± 0.3% after 8 h and maximum consumption rate of 0.043 g·L^−1^·h^−1^. In contrast, cultivation of *W. coagulans* BC99 in pH 7.00 medium (normal culture pH) accelerated glucose consumption markedly, achieving 21.4 ± 0.5% consumption within the same period and maximum rate of 0.12 g·L^−1^·h^−1^—significantly higher than under critical pH conditions (*p* < 0.05). For pH 7.00 medium (Figure 1E), protein nitrogen content in supernatant decreased gradually during cultivation, reaching maximum depletion of 6.7 ± 0.4%. Under critical pH, however, no significant change in supernatant protein concentration occurred throughout cultivation (*p* > 0.05), with residual concentration consistently higher than in pH 7.00. These results indicate *W. coagulans* BC99 exhibits significantly reduced capacity to utilize protein nitrogen sources under critical pH versus pH 7.00.

### 3.2. Illumina HiSeq mRNA Sequencing and Functional Classification of Unigenes

**Figure 2:**
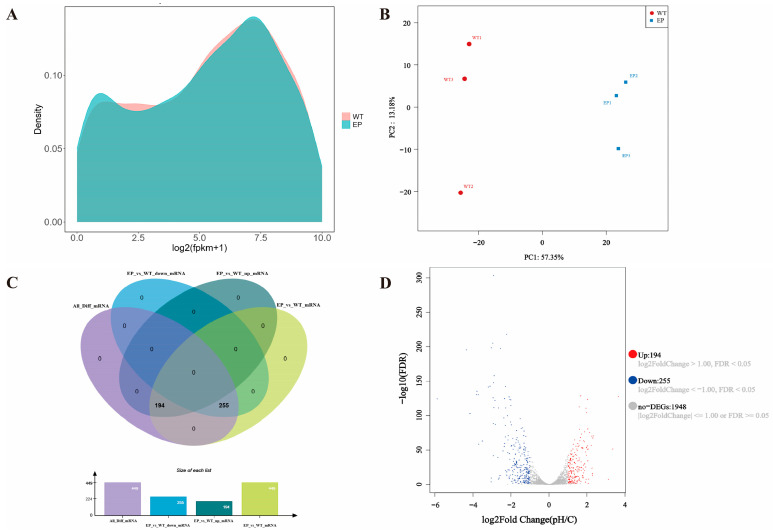
Transcriptome changes in *W. coagulans*. (**A**). expression density distribution. Red represents the control group (WT), green represents the low-pH treatment group (EP); (**B**). PCA based on gene expression quantity. Blue represents the control group (WT), red represents the low-pH treatment group (EP), and the numbers represent the score values of the principal components; (**C**). number of up and down genes; (**D**). volcano map of DEGs.

To further elucidate the influence of pH on the fermentation process in *W. coagulans*, Illumina RNA-Seq was employed to associate genes involved in key metabolic pathways with cell division. Figure 2A shows the probability density distribution of gene expression for all genes. The normal culture condition group (WT) and low-pH-treated (EP) groups exhibited highly congruent overall expression profiles, both displaying unimodal peaks centered at log_2_(FPKM + 1) ≈ 6. This distribution pattern confirms minimal global transcriptome restructuring. Nevertheless, systematic density reductions occurred across all expression ranges in EP relative to WT, indicating pH induced transcriptional suppression. Transcriptomes were compared between WT and EP groups. Principal component analysis (PCA) of WT and EP groups (Figure 2B) revealed sample separation in principal component space: PC1 captured 57.35% of total variance, indicating it represents the most significant biological differences—likely systematic transcriptional responses to pH stress. On the PC1-PC2 plane, WT and EP groups separated along PC1: EP clustered in the positive direction, while WT concentrated in the negative direction, confirming low pH remodels global expression profiles. PC2 explained 13.18% of variance but showed no significant group separation, suggesting it may reflect secondary biological processes or technical variation. The analysis of differential expression under experimental conditions identified 449 differentially expressed genes (DEGs) (Figure 2C; 194 upregulated, 255 downregulated). These DEGs’ expression patterns are visualized in the volcano plot (Figure 2D).

**Figure 3:**
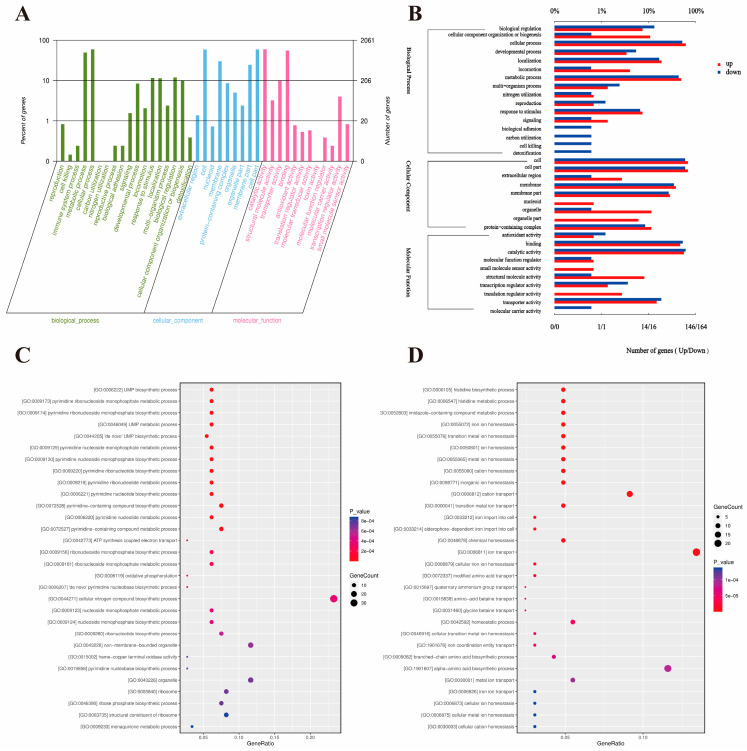
Differential expression of mRNA genes’ GO functional annotation and enrichment analysis. (**A**). The statistical chart of the secondary classification of GO; (**B**). the GO functional annotation diagram of significantly differentially expressed genes; (**C**). bubble plot of GO functional enrichment for significantly upregulated genes; (**D**). bubble plot of GO functional enrichment for significantly downregulated genes.

As shown in Figure 3A, differentially expressed genes are widely distributed across the three major categories of biological processes, cellular components, and molecular functions. Among them, at the biological process level, functions such as cellular processes, metabolic processes, biological regulation, and responses to stimuli are significantly enriched. Moreover, in all major categories, the number of downregulated genes is generally higher than that of upregulated genes, overall reflecting that cells may generally inhibit basic metabolism and regulatory activities under low-pH conditions, presenting an overall adaptive pattern dominated by expression downregulation (Figure 3B). Additionally, the vast majority of genes are annotated at the basic functional levels, such as catalytic activity, binding, cellular components, membrane structure, and cell and metabolic processes. Functional classification progresses to granular specificity, including nucleotide binding, ion binding, and intracellular localization (cytoplasm, membrane). This framework establishes that low pH-induced differential expression precisely localizes within metabolic, transport, and structural systems. Further detailed functional enrichment analysis (Figure 3C,D) reveals that this downregulation is not uniform globally but has a clear functional orientation: downregulated genes are significantly enriched in histidine biosynthesis, branched-chain amino acid synthesis, iron ion homeostasis maintenance, transition metal ion transport, and the transport of compatible solutes such as glycine and betaine, at the same time, upregulated genes are specifically enriched in de novo synthesis of purine nucleotides (such as UMP), purine nucleotide metabolism, oxidative phosphorylation, and ATP synthesis coupled electron transfer, as well as ribosome structure and ribosome composition.

**Figure 4:**
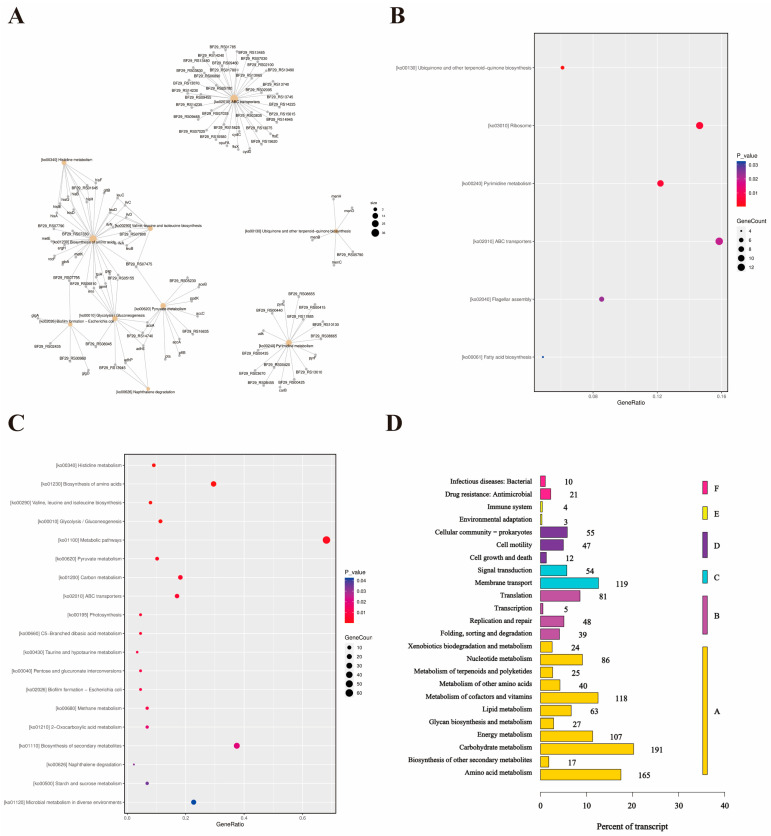
Differential expression of mRNA genes’ KEGG functional annotation and enrichment analysis. (**A**). KEGG pathway enrichment network diagram of significantly differentially expressed genes; (**B**). bubble chart of significantly upregulated genes in KEGG pathways; (**C**). bubble plot of gene enrichment for significantly downregulated KEGG pathways; (**D**). KEGG pathway classification statistics chart.

The subsequent KEGG pathway enrichment analysis revealed that *W. coagulans* exhibited systematic transcriptional reprogramming under low-pH stress (Figure 4A). The molecular adaptation mechanisms are mainly manifested in the following aspects: The low-pH environment first drives the stress response by affecting cell membrane integrity, protein stability, and enzyme activity. Upregulated genes predominantly enriched terpenoid-quinone biosynthesis (ko00130), ribosomal functions (ko03010), pyrimidine metabolism (ko00240), ABC transporters (ko02010), flagellar assembly (ko02040), and fatty acid biosynthesis (ko00061). These pathways exhibited high gene representation with statistical significance (Figure 4B). Metabolic pathways exhibited significant suppression, encompassing histidine metabolism (ko00340), multiple amino acid biosynthesis (ko01230, ko00290), central carbon metabolism (glycolysis/gluconeogenesis ko00010; pyruvate metabolism ko00620; carbon metabolism ko01200), photosynthesis (ko00195), biofilm formation (ko02026), and secondary metabolite biosynthesis (ko01110). These downregulated pathways demonstrated variable gene representation with substantial enrichment in specific categories (Figure 4C). From the functional classification of the overall transcripts, the transcriptional response of *W. coagulans* was highly concentrated in metabolism-related processes. Among them, the transcripts of carbohydrate metabolism and amino acid metabolism accounted for the highest proportion, followed by basic physiological processes, such as membrane transport, energy metabolism, translation, and lipid metabolism. Other categories, such as nucleotide metabolism, cofactor metabolism, and functions related to genetic information processing, also accounted for a definite proportion (Figure 4D).

### 3.3. Differential Gene Expression During Division and Growth Under pH Stress

**Figure 5:**
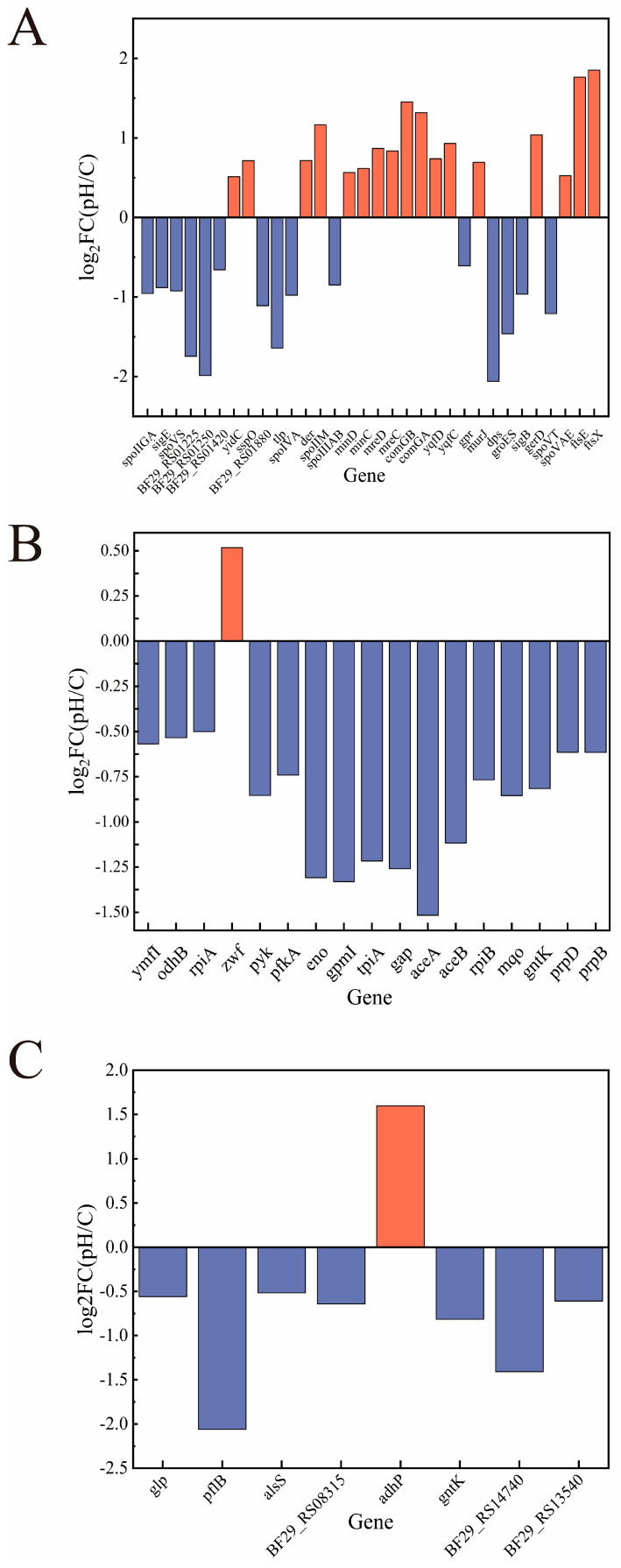
Expression of related pathway genes under pH stress: (**A**). division process, (**B**). energy-metabolism process, (**C**). acid production process. Blue indicates a decrease, while red indicates an increase.

In the balance of the core life activities of cell division and spore formation, low-pH stress significantly inhibits the spore formation pathway and promotes the activation of the cell division mechanism. Figure 5A demonstrates significant downregulation of key sporulation genes. Early sporulation σ factor *sigE* and its protease-encoding target *spoIIGA* show inhibited expression, blocking initiation signals. Conversely, cell division genes display marked upregulation. The expression of *ftsX* and *ftsE*, the core components of the divisome, is significantly enhanced, which directly drives septum formation and cell division. Under acidic stress, the Min system (*minD*, *minC*) and the Mre system (*mreD*, *mreC*) exhibit upregulation. The Min system regulates cell polarity and division site selection, while the Mre system maintains cell shape [17,18]. This coordinated response ensures accuracy in cell division and stability in cell morphology. In addition, the *comG* operon (*comGB*, *comGA*) involved in competence development and possibly associated with DNA uptake is significantly activated. In a low-pH environment, bacteria suppress energy-intensive sporulation.

Secondly, matching the above-mentioned phenotypic transformation, the cell’s energy-metabolism network is reprogrammed, manifested as the downregulation of glycolysis and some branch metabolic pathways, which may be related to the adjustment of energy-production efficiency and the change in carbon-flow direction (Figure 5B). The expression of multiple key transcripts of the glycolytic (EMP) pathway decreases consistently, including *pfkA*, *gap*, *tpiA*, *gpmI*, *eno*, and *pyk*, and the amount of pyruvate produced through the classical glycolytic pathway decreased. The gene encoding the core subunit E2 of the dihydrolipoyl succinyltransferase *odhB* in the tricarboxylic acid cycle (TCA) and the genes *rpiA* and *rpiB* encoding the only reversible isomerization reaction catalyst in the pentose phosphate pathway are also downregulated. Low pH activates the OxyR/SoxRS regulatory system through oxidative stress, inducing the upregulation of *zwf*, and redirecting the carbon flow from the energy-producing TCA to the pentose phosphate pathway that generates NADPH [19]. Notably, the key genes *aceA* and *aceB* of the glyoxylate cycle are severely inhibited, and bacteria restrict C2 substrate utilization (e.g., acetate). This metabolic adaptation minimizes acidogenic intermediate accumulation, *prpB* and *prpD* related to propionate metabolism and pyruvate formate lyase pflB are downregulatedto 24% of the control [20]. The change in energy metabolism meets ATP demand, balances reducing power, and prevents intracellular acidification from worsening.

In terms of lactate metabolism and acid-stress response, bacteria initiate a series of adaptive programs that respond to the internal-environment pH, protect macromolecules, and cell structures. This is shown in Figure 5C, the typical lactate dehydrogenase gene *BF29_RS14740* was downregulated by 63%, and the key enzyme pflB for mixed-acid fermentation was downregulated by 76%, thereby inhibiting the branch of acidic products caused by pyruvate. The expression of acetolactate synthase alsS is downregulated to about 75% of the control, further restricting the conversion of pyruvate to acetolactate [21]. The genes directly responding to acid stress show differential expression: the gene *dps* encoding the DNA-binding protective protein is significantly downregulatedwhich may be a regulatory mode different from oxidative stress under specific acid stress; the molecular chaperone *groES* is down-regulated, suggesting that protein-folding stress may not be the core contradiction at this stage. However, the strong upregulation of the alcohol dehydrogenase gene *adhP* is noteworthy. In addition, genes such as *murJ* involved in cell-membrane lipid synthesis and *yidC* and *der* involved in membrane-protein insertion are upregulated. The global stress-regulatory factor sigB is downregulated. The analysis of the transcriptome under low-pH stress reveals the multi-level and coordinated regulation of cell division, spore formation, energy metabolism, and acid-stress-related pathways by this bacterium in an acidic environment to maintain survival and proliferation.

### 3.4. RT-qPCR Validation

**Figure 6:**
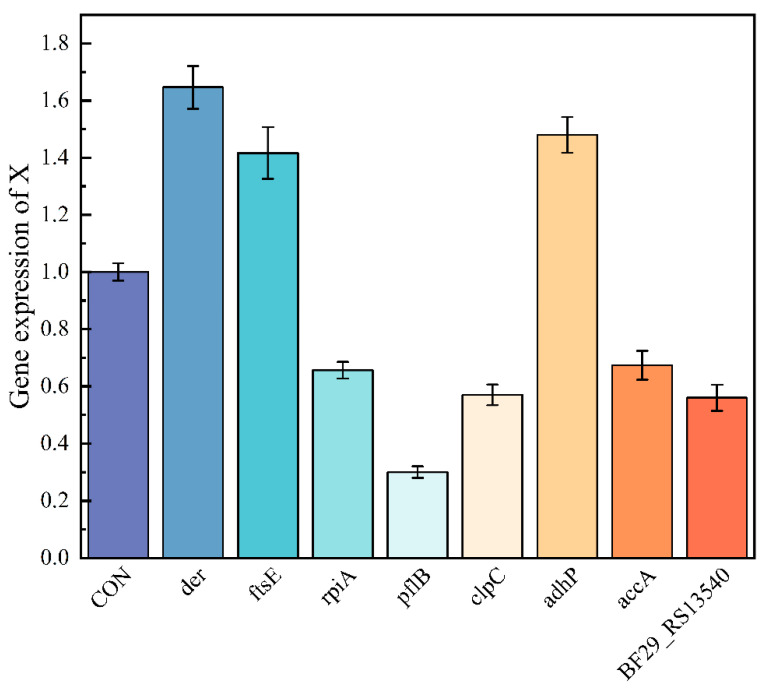
RT-qPCR verification of some differentially expressed genes under pH stress.

Figure 6 shows the relative expression levels of multiple target genes. The expression level of the *der* gene was significantly higher than that of all other genes, with its expression level being approximately 4 to 6 times that of the other genes, indicating that it is a highly expressed gene. The expression of *rpiA* and *pflB* genes was at a moderate level. The expression levels of *clpC*, *adhP* and *accA* genes were low and close to each other, representing about 10–15% of the expression level of *der*. The expression level of *BF29_RSI3540* was the lowest among all tested genes, and its column height was only about 5% of *der*, showing a very low expression state.

Among the differentially expressed genes described in the Results section, eight were selected for RT-qPCR validation. The RT-qPCR results confirmed the differential expression patterns of these genes, supporting the reliability of the RNA-seq data obtained in this study.

## 4. Discussion

The pH value plays a critical role in bacterial culture processes. This study investigated the effects of critical pH conditions on cell division in *W. coagulans* BC99 and analyzed transcriptome-level gene expression changes under low-pH conditions. This study provides a theoretical basis for improving the fermentation performance of *W. coagulans* by revealing the changes in energy consumption and metabolism, including carbon and nitrogen sources, and analyzing multiple potential functional genes related to cell division and metabolism.

Some studies indicate that medium acidification affects fermentation-related enzymes in *W. coagulans*, reducing ATP production efficiency and metabolic intermediate synthesis [22]. This impairment compromises intracellular substance synthesis, leading to inhibited cell proliferation. Pyruvate dehydrogenase in glycolysis can directly regulate FtsZ protein. Decreased pyruvate levels diminish pyruvate dehydrogenase activity, disrupting divisome ring formation and altering cellular division states [23]. These observations suggest that critical pH conditions modulate division proteins through perturbations in glucose metabolism. Research suggests that under carbon source metabolism inhibition, bacteria may metabolize amino acids to generate energy, ensuring viability in adverse conditions [24]. Although *W. coagulans* maintains viability at critical pH, division is inhibited and carbon source consumption decreases significantly. Consumed amino acids may support both intracellular pH homeostasis and energy production. By utilizing amino acids, bacteria adapt their proliferative states, and targeted amino acid supplementation could favor division at critical pH.

The physiological response to acid stress is initially reflected in enhanced protein synthesis capacity and increased ribosome biogenesis. Upregulation of *rpsP* and *rpsB* expression increases translational efficiency, promotes 30S ribosomal subunit assembly, and supports rapid cell proliferation under acidic conditions [25,26]. Concurrently, *rplQ* and *rplM* of the 50S ribosomal subunit were also upregulated, further promoting protein synthesis. Ribosomal protein upregulation enhances bacterial capacity to produce repair factors and division-specific molecules. This offsets suppressed translational activity under low-pH. Such biosynthetic support is essential for cell division [27]. It has been shown that the proper assembly and functional integrity of the Fts complex are essential for successful cytokinesis during cell division. Both ftsE and ftsX were upregulated to varying degrees, and the proteins encoded by them are components of the Fts complex that promote division body formation and membrane remodeling, especially during the later stages of septation, in line with previous reports [28]. Under low-pH conditions, the structural integrity of the cell membrane and cell wall is compromised; bacteria counteract this by upregulating Fts complex-related genes to reinforce the division machinery, restore membrane architecture, and ensure the fidelity of the division process [29]. Additionally, ftsA—which acts as a stabilizing factor for the FtsZ ring—supports orderly progression of cytokinesis through its enhanced expression.

When environmental acidity reaches the critical threshold (pH < 4.20), global transcriptional reprogramming is initially characterized by a significant downregulation in the expression of the ATP operon (including atpA, atpD, and atpG) and the αβ subunit (BF29_RS09985), resulting in reduced synthesis efficiency of the F_0_F_1_-ATP synthase complex [30]. This transcriptional pattern aligns with the principle of energy conservation under stress. In *B. subtilis*, the C-terminal regulatory domain of the ε subunit enhances ATP-dependent H^+^ pumping activity, which is critical for maintaining membrane potential under energy-limiting conditions [31]. To counteract the detrimental effects of acidic environments on cellular functions, bacteria upregulate specific genes to enhance proton pump activity, thereby maintaining proton motive force (PMF) homeostasis and ensuring energy conservation [32]. Bacterial adaptation involves enhanced proton pumping via the respiratory chain. Specifically, terminal oxidase genes *qox* operon and *cydA* show upregulation. This enhances proton extrusion, restoring transmembrane electrochemical gradients and sustaining ATP synthesis [33,34]. It was also found that bacteria induced changes in the expression of genes involved in quinone electron carrier biosynthesis (menB, menC, menD, menH) and cytochrome assembly heme biosynthesis (hemC), presumably supporting the high metabolic demands of the enhanced respiratory system. Concurrently, the induction of changes in molecular chaperones (e.g., dnaK, dnaJ) [35,36] and membrane lipid-remodeling enzymes (e.g., accA, accC), which contribute to protein stability and membrane integrity [37,38], are induced. Enhanced magnesium uptake via CorA helps stabilize intracellular magnesium levels, which is critical because inhibition of the division process is synergistically affected by changes in membrane stability and ion homeostasis [39].

In addition, under conditions of H^+^-mediated competitive displacement, essential metal cofactors such as Cu^2+^ are depleted, leading to diminished activity of copper-containing cytochrome oxidases, and weakening the energy supply network required for division and contraction. Furthermore, conformational rearrangement within the C-terminal domain of the β subunit disrupts the functional coupling between the proton translocation channel and the catalytic sites, thereby enhancing the maintenance of membrane potential (ΔΨ) [40]. The PhoPR two-component system functions as a central regulatory node. The PhoP response regulator represses *gltA*, redirecting carbon flux toward glycolytic pathways, while simultaneously activating the degradation pathway of rpsU (encoding ribosomal protein S21), which reduces *ftsZ* mRNA translation efficiency. PhoP also inhibits the activity of the peptidoglycan hydrolase RipA, delaying septal cleavage [41,42]. This multi-target regulatory strategy ensures that cells prioritize entry into a dormant state under energy-limiting conditions. Transcriptomic data also show significant upregulation of *gltB* (encoding glutamate synthase), suggesting enhanced nitrogen assimilation capacity to support intracellular pH homeostasis [43]. Furthermore, the energy supply system plays a pivotal role in sustaining cell division under low-pH stress. The genes *aceA* and *aceB* encode key enzymes in the tricarboxylic acid (TCA) cycle, regulating carbon flux to generate reducing equivalents (such as NADH) and biosynthetic precursors essential for cellular division. Under acidic conditions, bacteria require additional energy to maintain proton motive force, power repair systems, and sustain biosynthesis processes [44]. The coordinated upregulation of phosphotransferases and iron/manganese superoxide dismutase ensures a continuous and stable energy supply for these high-energy-demanding processes.

Compared to the highly acidic metabolites—such as formate and acetate—produced via the mixed-acid fermentation pathway mediated by pyruvate formate lyase (PflB) and phosphotransacetylase (Pta), lactate accumulation exerts a comparatively minor effect on the intracellular proton gradient [45,46]. The metabolic shift toward lactate production not only prevents energy collapse and impairment of the respiratory chain due to dissipation of the proton motive force (PMF), but also substantially reduces secondary cellular damage arising from endogenous acidification. Concurrently, bacteria downregulate key metabolic genes (e.g., *pflB*, *adhE*, *pta*) to inhibit the production of acidic metabolites such as formate and acetate, mitigating the progression of intracellular acidification at its source [47]. Under acid stress, bacteria thus utilize lactate accumulation to maintain energy homeostasis while minimizing pH perturbations.

When acid stress reaches a critical threshold, cells initiate a profound metabolic contraction response. Lactate dehydrogenase (LDH) expression is markedly downregulated, and the transcriptional regulator SirA is concurrently suppressed. Key mixed-acid fermentation genes (*pflB*, *pflA*, *adhE*) and acetic acid biosynthesis genes (*pta*) are globally repressed. This coordinated transcriptional silencing limits organic acid production, thereby minimizing proton influx and reducing reliance on ATP-dependent proton extrusion systems such as F_0_F_1_-ATP synthase, thus preserving intracellular pH homeostasis [48]. During this phase, bacteria selectively inhibit glycolytic fermentation branches while redirecting glucose flux toward non-acidogenic pathways, including the pentose phosphate pathway, to conserve energy and sustain cellular viability. Concurrent downregulation of amino acid biosynthesis operons (e.g., *his*, *ilv*, *leu*) further diminishes the energetic burden of macromolecular synthesis, facilitating entry into a state of deep metabolic dormancy and enabling the maintenance of minimal essential energy expenditure [49].

At the molecular level, the bidirectional regulation of SirA reveals the hierarchical organization of the bacterial stress response network. Under mild stress conditions, SirA functions as a positive regulator of the lactate dehydrogenase (LDH) gene cluster, enhancing transcription through specific promoter binding and recruitment of RNA polymerase σ factors [50]. However, when environmental pH exceeds physiological thresholds, global stress regulators such as σᴮ suppress SirA activity via competitive DNA binding or epigenetic modifications, reorganizing the regulatory hierarchy to ensure a timely transition to a conservative survival mode. The expression dynamics of core glycolytic enzymes—such as glyceraldehyde-3-phosphate dehydrogenase (*Gap*) and enolase (*eno*)—are tightly coordinated with those of fermentation-related genes, indicating that lactate metabolism is intricately integrated within the framework of central carbon flux regulation [51]. When NADPH demand from the pentose phosphate pathway exceeds ATP generation via glycolysis, key metabolic effectors—including ATP/ADP and NADH/NAD^+^ ratios—exert negative feedback on glycolytic flux [52]. This modulation fine-tunes carbon allocation at the pyruvate branch point and balances the competitive utilization of alternative fermentation pathways. Such a metabolic shift enables cells to precisely regulate metabolic flow, minimize futile energy expenditure, and maximize the probability of survival under adverse conditions.

## 5. Conclusions

Our findings demonstrate that *W. coagulans* orchestrates acid stress tolerance through the synergistic regulation of energy-metabolism–cell-division coupling and cellular homeostasis. Under low-pH conditions, the bacterium achieves strategic ATP conservation via repression of the F_0_F_1_-ATP synthase and optimization of ABC transporter activity, while cyclopropane fatty acid synthesis enhances membrane rigidity at the expense of divisome substrate transport efficiency. Transcriptional rewiring mediated by the PhoPR signaling pathway coordinates ribosomal protein degradation with redistribution of metabolic flux, thereby establishing a proteostatic bottleneck that limits the availability of key division proteins. The collateral consequences of proton gradient dissipation—including metal cofactor displacement and oxidative damage originating from the electron transport chain—are counteracted by CorA-mediated magnesium chelation and robust ROS detoxification systems. Critically, this study identifies quorum sensing-mediated biofilm formation as an energy-intensive survival adaptation that paradoxically exacerbates cell division arrest by triggering SulA-dependent blockade of Z-ring maturation. However, batch culture conditions may not fully represent natural or industrial environments. Continuous culture can be used for further research. Despite the limitations, the findings have practical relevance. This study provides molecular insights into how *W. coagulans* preserves viability under acid stress, offering a theoretical basis for optimizing its performance in industrial applications.

## Data Availability

The original contributions presented in this study are included in the article. Further inquiries can be directed to the corresponding authors.

## Abbreviations

The following abbreviations are used in this manuscript:

KEGG	Kyoto Encyclopedia of Genes and Genomes
GO	Gene Ontology
